# Comprehensive Analysis of Virulence Genes, Antibiotic Resistance, Biofilm Formation, and Sequence Types in Clinical Isolates of *Klebsiella pneumoniae*

**DOI:** 10.1155/cjid/1403019

**Published:** 2024-12-19

**Authors:** Mohsen Nazari, Jaber Hemmati, Babak Asghari

**Affiliations:** Department of Microbiology, School of Medicine, Hamadan University of Medical Sciences, Hamadan, Iran

**Keywords:** antibiotic resistance, biofilm, *Klebsiella pneumoniae*, molecular epidemiology, virulence factors

## Abstract

**Background:** The rise in multidrug-resistant pathogens poses a formidable challenge in treating hospital-acquired infections, particularly those caused by *Klebsiella pneumoniae*. Biofilm formation is a critical factor contributing to antibiotic resistance, enhancing bacterial adherence and persistence. *K. pneumoniae* strains vary in virulence factors, influencing their pathogenicity and resistance profiles. This study aimed to comprehensively analyze virulence factors, antibiotic resistance patterns, and biofilm formation in clinical isolates of *K. pneumoniae* from Hamadan hospitals. Moreover, the study explored the molecular epidemiological relationships among isolates using multilocus sequence typing (MLST) to uncover the genetic diversity associated with resistance and virulence.

**Materials and Methods:** Between December 2022 and April 2024, 402 *K. pneumoniae* isolates were collected from clinical samples, including urine, tracheal aspirates, blood, wounds, and abscesses, in teaching hospitals in Hamadan. Initial culturing was performed on blood agar and MacConkey agar, and isolates were identified using biochemical tests. Antimicrobial susceptibility testing followed CLSI, employing the Kirby–Bauer disk diffusion method with 10 antibiotics. Biofilm formation was assessed using the microtiter plate method, and virulence genes were detected by PCR. MLST analysis was conducted on 10 selected isolates based on their virulence gene profiles and resistance patterns.

**Result:** Of the 456 clinical isolates analyzed, 402 (88.15%) were identified as *K. pneumoniae*, predominantly isolated from tracheal samples (251/402, 62.44%), followed by urine (105/402, 26.12%), blood (30/402, 7.46%), wounds (15/402, 3.73%), and abscesses (1/402, 0.25%). Antibiotic resistance rates revealed high resistance to cefepime (356/402, 88.55%), imipenem (345/402, 85.82%), and ceftazidime (305/402, 75.87%), while resistance to amikacin (165/402, 41.04%) and piperacillin–tazobactam (75/402, 18.65%) was comparatively lower. Biofilm formation varied among the isolates, with 17/402 (4.22%) forming strong biofilms, 104/402 (25.87%) moderate biofilms, 180/402 (44.78%) weak biofilms, and 101/402 (25.12%) showing no biofilm production. Virulence gene analysis indicated high prevalence rates for *mrkD* (396/402, 98.50%), *fimH1* (351/402, 87.31%), and *entB* (402/402, 100%), while genes like *irp-1* (151/402, 37.56%) and *irp-2* (136/402, 33.83%) were less common, and *hylA* and *cnf-1* were absent. MLST analysis of 10 selected isolates identified sequence types ST147 (5/10, 50%), ST11 (3/10, 30%), and ST15 (2/10, 20%).

**Conclusion:** *K. pneumoniae* demonstrates notable biofilm-associated antibiotic resistance, supported by a significant association with XDR strains, along with a diverse array of virulence gene profiles. The study underscores the importance of understanding molecular epidemiology for effective management of hospital infections, emphasizing the need for targeted surveillance and infection control measures.

## 1. Introduction

*Klebsiella pneumoniae* is a common opportunistic pathogen frequently responsible for nosocomial infections such as pneumonia, meningitis, and infections of the bloodstream and urinary tract [1]. Recently, *K. pneumoniae* has been categorized into two distinct types: classical *K. pneumoniae* (cKp) and hypervirulent *K. pneumoniae* (HvKp), based on their differences in virulence [2, 3]. HvKp differs from cKp primarily due to its increased potential for metastatic dissemination and multisite infections [4]. Phenotypically, HvKp is characterized by hypermucoviscous colonies resulting from excessive capsule production, while genotypically, it is identified by specific capsular types and iron-acquisition genes [5, 6].

*K. pneumoniae* predominantly causes infections in the respiratory system, urinary tract, bloodstream, and wounds, with a significant prevalence reported in Asia [7–9]. This bacterium's high virulence is often associated with capsular serotype K1 or K2, which exhibits a hypermucoviscosity phenotype due to the presence of a K1-specific gene (*magA*) and a plasmid-borne gene (*rmpA*) [10]. Additional virulence factors in *K. pneumoniae* include fimbrial and nonfimbrial adhesion genes such as *mrkD*, *KPN*, and *ycfM* [11]. The *mrkD* gene encodes an adhesin that facilitates the bacterium's attachment to the extracellular matrix [12]. The *mrkA* gene, on the other hand, encodes the major pilin subunit of Type 3 fimbriae, which is important for the structural formation of these fimbriae. Another critical factor is the FimH protein, a subunit of the Type 1 fimbrial adhesin, which plays a significant role in kidney infections caused by *K. pneumoniae* [13].

To sustain infection, *K. pneumoniae* requires efficient iron acquisition [14]. Molecular epidemiology studies have revealed that hypervirulent strains possess multiple iron uptake systems [15]. These include enterobactin (ENT), a catecholate siderophore, aerobactin (a hydroxamate siderophore with receptors encoded by *iutA* genes), yersiniabactin (*ybtS*), a phenolate siderophore similar in structure to Ent, and Kfu, which facilitates ferric iron uptake. These systems are more commonly found in hypervirulent strains of *K. pneumoniae* [16–18].

Genotyping is crucial for identifying cases or outbreaks caused by *K. pneumoniae* and tracking the sources and spread of infections [19]. Common genotyping methods for genotyping *K. pneumoniae* include pulsed field gel electrophoresis (PFGE), multiple-locus variable number tandem repeat analysis (MLVA), and multilocus sequence typing (MLST), with MLST being the most widely used [20]. The MLST scheme for *K. pneumoniae*, developed in 2005, has been used globally to study the diversity and epidemiology of clinical isolates, leading to the identification of various clones with distinct virulence or drug resistance characteristics [21, 22]. Understanding the relationships between *K. pneumoniae* virulence factors, sequence types (STs), and the infections they cause is critical for controlling transmission. This study examines these connections.

## 2. Materials and Methods

### 2.1. Patients and Specimens

Between December 2022 and April 2024, a total of 456 isolates were consecutively collected from all eligible clinical samples identified in various wards of teaching hospitals in Hamadan. The isolates were obtained through routine diagnostic procedures conducted in hospital laboratories during the study period.

The bacterial isolates were obtained from clinical samples, including urine, tracheal aspirates, blood, and wound swabs. The initial culturing of the samples was performed on blood agar (Merck, Germany) and MacConkey agar (Merck, Germany) in hospital laboratories. Following bacterial growth, the isolates were subcultured and sent to the microbiology department at Hamadan University of Medical Sciences. After an overnight incubation at 37°C, a series of microbiological and biochemical tests were conducted. These included Gram staining, observation of colony morphology, triple sugar iron (TSI) agar reaction, citrate utilization, urease test, indole production, methyl red/Voges–Proskauer (MR/VP) test, and motility testing. These tests facilitated a detailed phenotypic characterization of the isolates, consistent with *K. pneumoniae* [23]. *K. pneumoniae* ATCC 700603 served as the reference strain for species identification [24].

### 2.2. Examination of Biofilm Formation

To investigate biofilm formation and phenotypic antibiotic resistance patterns, the microtiter plate method was employed [25]. A 96-well microplate was prepared by adding 200 μL of a bacterial suspension (10^6^ CFU/mL) cultured in tryptic soy broth (TSB) (Merck, Germany) medium with 0.25% glucose to each well. The plate was then incubated at 37°C for 24 h. The negative control consisted of 200 μL of TSB medium without bacterial inoculation, added to the corresponding wells and incubated under the same conditions, while *K. pneumoniae* ATCC 700603 served as the positive control for strong biofilm production [25]. After incubation, the wells were emptied and washed three times with phosphate-buffered saline (PBS, pH 7.2). To fix the biofilm, 200 μL of 99% methanol was added to each well and allowed to sit at 25°C for 15 min. The methanol was removed, and the wells were stained with 200 μL of 2% crystal violet for 5 min. Excess stain was rinsed off with distilled water, and the plate was air-dried at 25°C. Subsequently, 180 μL of 33% (v/v) glacial acetic acid was added to fully dissolve the remaining stain. The absorbance of each well was measured at 630 nm using an ELISA reader (Tecan, Switzerland). Each isolate's biofilm formation assay was performed in triplicate. Biofilm formation was assessed by comparing the optical density (OD) of each well to that of the control well, and results were categorized as follows [26]:

(1) $$\begin{array}{c} \text{Strong biofilm}:\text{OD}>4\times \text{OD cut}-\text{off value}\left(\text{ODc}\right),\\ \text{Moderate biofilm}:2\times \text{ODc}<\text{OD}\leq 4\times \text{ODc},\\ \text{Weak biofilm}:\text{ODc}<\text{OD}\leq 2\times \text{ODc},\\ \text{Nonbiofilm producer}:\text{OD}\leq \text{ODc}. \end{array}$$

ODc is the threshold value used to differentiate between biofilm production categories and is calculated as the mean OD of the negative control plus three standard deviations.

### 2.3. Antimicrobial Susceptibility Testing

Antimicrobial susceptibility testing was performed using the Kirby–Bauer disk diffusion method, following the Clinical and Laboratory Standards Institute (CLSI, 2024) guidelines [27]. Mueller-Hinton agar plates were used to test a panel of 10 antibiotic discs. These included amikacin (30 μg) and gentamicin (10 μg) from the aminoglycoside family, ciprofloxacin (30 μg) from the fluoroquinolone class, and three cephalosporins: cefepime (30 μg), a 4th-generation cephalosporin, cefoxitin (30 μg), a 2nd-generation cephalosporin, and ceftazidime (30 μg), a 3rd-generation cephalosporin. Imipenem (10 μg) was also tested, representing the carbapenem class. Additionally, piperacillin–tazobactam (10/100 μg) and ampicillin–sulbactam (10/10 μg), both beta-lactam/beta-lactamase inhibitor combinations, as well as trimethoprim–sulfamethoxazole (23.75/1.25 μg), were included in the panel. All antibiotic discs used in the study were sourced from Mast Group Ltd., UK. Furthermore, *K. pneumoniae* isolates nonsusceptible to at least one agent in three or more distinct antimicrobial classes were defined as multidrug-resistant (MDR), while those nonsusceptible to at least one agent in six or more different antimicrobial categories were defined as extensively drug-resistant (XDR) [28].

### 2.4. DNA Extraction

Total DNAs of *K. pneumoniae* isolates were extracted by the boiling method [29]. Briefly, 3–5 colonies from an overnight bacterial culture were suspended in 500 μL of sterile distilled water. The suspension was boiled for 30 min and then centrifuged at 14, 000g for 5 min to pellet the cell debris. The supernatant containing the extracted DNA was collected and stored at −20°C. The quantity and quality of the extracted DNA were assessed using a NanoDrop ND-1000 spectrophotometer (Biocompare, San Francisco, USA) and confirmed by 1.5% agarose gel electrophoresis.

### 2.5. PCR Detection of Virulence-Associated

The genes encoding Type 1 and Type 3 adhesins (*fimH-1*, *mrkD*), FimH-like adhesin (*kpn*), outer membrane lipoprotein (*ycfM*), catecholate siderophore receptor (*iroN*), ENT biosynthesis (*entB*), aerobactin receptor (*iutA*), yersiniabactin receptor (*fyuA*), yersiniabactin biosynthesis (*irp-1*, *irp-2*, *ybtS*), serum resistance-associated outer membrane lipoprotein (*traT*), regulator of mucoid phenotype A (*rmpA*), mucoviscosity-associated gene A (*magA*), *α*-hemolysin (*hlyA*), and cytotoxic necrotizing factor-1 (*cnf-1*) were detected using PCR (the study primers are listed in Table 1) [17].

### 2.6. Serotyping and MLST for *K. pneumoniae* Isolates

The MLST analysis followed the Pasteur scheme, targeting seven housekeeping genes of *K. pneumoniae*. PCR was conducted in a 50-μL reaction mixture containing 25 μL of 2x PCR Master Mix (Cinaclone Co., Iran), 1 μL of each primer (10 μM), and 2 μL of sample DNA. The PCR cycling conditions were as follows: an initial denaturation at 94°C for 2 min, followed by 35 cycles of denaturation at 94°C for 20 s. The annealing temperatures were set at 50°C for most genes, except for *tonB* (45°C) and *gapA* (60°C), with an annealing time of 30 s. This was followed by a 30-s extension at 72°C, and a final extension step at 72°C for 5 min. PCR was performed using a Bio-Rad Thermal Cycler (Bio-Rad Inc., United States) [21].

The amplified gene products were visualized on a 1% agarose gel stained with ethidium bromide, and their sizes were compared to a GeneRuler 1 kb plus DNA ladder (Bioneer Co., Korea). Images of the gel were captured using a gel documentation system (MWG-Biotech, Germany). Sequencing of the genes was done using both forward and reverse primers, provided by Macrogen. STs were determined by referencing the MLST database for *K. pneumoniae* at https://bigsdb.pasteur.fr/klebsiella/.

### 2.7. Statistical Analysis

Serotyping and MLST of *K. pneumoniae* isolates were conducted using SPSS 19 software for data analysis (Chicago, IL, USA). Student's *t*-test was employed to analyze numerical data, with statistical significance set at *p* < 0.05. The sensitivity and specificity of phenotypic methods were assessed against PCR, considered the gold standard method, using the Chi-square test.

## 3. Results

### 3.1. Isolation of *K. pneumoniae* From Various Clinical Samples

**Figure 1:**
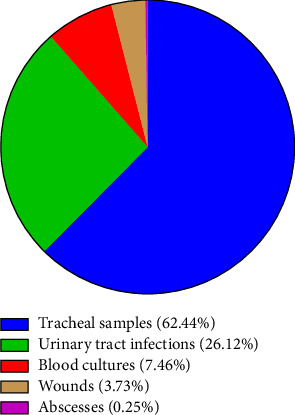
Frequency of *Klebsiella pneumoniae* in different clinical isolates.

In this study, a total of 456 isolates were obtained from various clinical samples. Among these, 402 isolates (88.15%) were identified as *K. pneumoniae* using differential tests. The distribution of these isolates by gender showed that 216 (53.73%) were from women and 186 (46.26%) from men. The statistical analysis showed a significant relationship exists between MDR/XDR profiles and gender, suggesting that men are more prone to developing drug-resistant infections. Also, a significant relationship exists between MDR/XDR and age groups, with younger children (0–5 years) and elderly individuals (60 years and older) exhibiting higher susceptibility to infections caused by MDR/XDR due to their developing or weakened immune systems, while those in the middle age groups generally show a lower prevalence of resistant *K. pneumoniae* infections (Table 2). Regarding clinical sample origins, the isolates were sourced as follows: 251 (62.44%) from tracheal samples, 105 (26.12%) from urinary tract infections, 30 (7.46%) from blood cultures, 15 (3.73%) from wounds, and 1 (0.25%) from abscesses (Figure 1).

### 3.2. Antibiotic Resistance Pattern in Clinical Isolates of *K. pneumoniae*

Out of 402 *K. pneumoniae* isolates obtained from various clinical samples, resistance was observed as follows: 242 (60.19%) isolates were resistant to cefoxitin, 275 (68.40%) to ciprofloxacin, 305 (75.87%) to ceftazidime, 345 (85.82%) to imipenem, 276 (68.65%) to gentamicin, 165 (41.04%) to amikacin, 75 (18.65%) to piperacillin–tazobactam, 122 (30.34%) to ampicillin–sulbactam, 252 (62.68%) to trimethoprim–sulfamethoxazole, and 356 (88.55%) to cefepime. Notably, cefepime showed the highest resistance frequency, while amikacin exhibited the lowest. Further detailed information, including data based on gender, can be found in Table 3.

### 3.3. Biofilm Formation Ability in Clinical Isolates of *K. pneumoniae*

**Figure 2:**
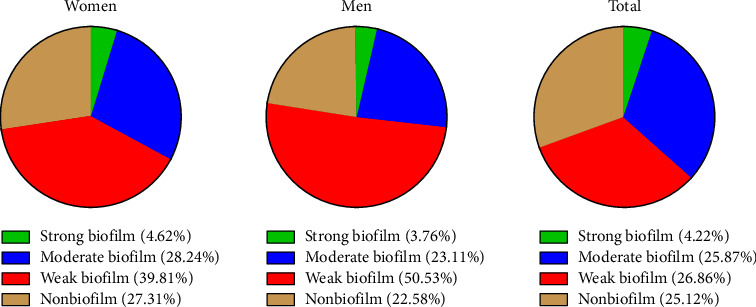
Biofilm formation classification based on optical density (OD) values of *Klebsiella pneumoniae* isolates from women and men samples.

**Figure 3:**
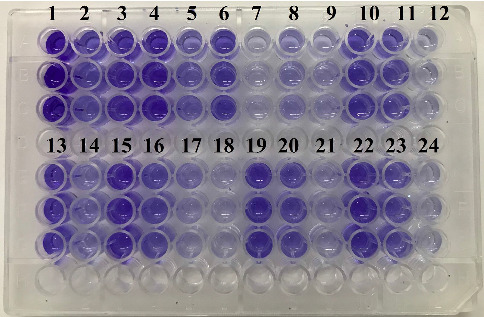
Results demonstrating the biofilm-forming ability of *Klebsiella pneumoniae* isolates, presented in triplicate (Columns 1–10 and 13–22). Positive controls are shown in Columns 11 and 23, and negative controls are represented in Columns 12 and 24.

Among the 402 *K. pneumoniae* isolates collected from diverse clinical samples, 17 (4.22%) isolates demonstrated strong biofilm formation, 104 (25.87%) isolates displayed moderate biofilm formation, and 180 (26.86%) isolates showed weak biofilm formation, and 101 (25.12%) isolates exhibited no detectable biofilm formation capability (Figures 2 and 3).

### 3.4. The Distribution Pattern of Virulence Genes in Clinical Isolates of *K. Pneumonia*

Among 402 *K. pneumoniae* isolates sourced from diverse clinical samples, the prevalence rates of specific genes were determined as follows: *fimH1* was found in 351 isolates (87.31%), *mrkD* in 396 isolates (98.50%), *kpn* in 245 isolates (60.94%), *ycfM* in 386 isolates (96.01%), and *entB* in all isolates (100%). Other notable genes included *iutA* (182 isolates, 45.27%), *irp-1* (151 isolates, 37.56%), *irp-2* (136 isolates, 33.83%), *ybtS* (254 isolates, 63.18%), and *fyuA* (235 isolates, 58.45%). Gel electrophoresis results for *iutA* and *mrkD*, obtained from PCR analysis, are shown in Supporting File 1. Less common genes such as *magA* and *iroN* were found in only 11 (2.73%) and 2 isolates (0.49%), respectively. No isolates tested positive for *hylA* and *cnf-1* (Table 4).

### 3.5. Typing Isolates Using the MLST Method

Out of 402 study isolates, 10 were selected for ST analysis based on their high resistance to multiple antibiotics and the presence of a higher number of virulence factors. These isolates were chosen to further investigate the potential correlation between their virulence gene content and antibiotic resistance profiles. Among these 10 isolates, the most diverse STs observed was ST147, represented by 5 isolates (5/10, 50%). This was followed by ST11, found in 3 isolates (3/10, 30%), and ST15, present in 2 isolates (2/10, 20%).

### 3.6. Association of Antibiotic Resistance, Biofilm Formation, Virulence Genes, and STs

This study analyzed 214 isolates (53.23%) categorized as MDR and 53 isolates (13.18%) as XDR. No significant association was found between biofilm formation and MDR isolates (*p* > 0.05), whereas a significant correlation was observed between biofilm formation and XDR isolates (*p* < 0.001). The distribution of biofilm formation among these isolates is presented in Table 5.

Furthermore, the relationship between the presence of virulence genes and the classification of XDR and MDR isolates is presented in Table 6. Notably, a positive correlation was observed between the intensity of biofilm formation and the abundance of virulence genes. Table 6 demonstrates that isolates with stronger biofilm formation tend to possess a greater number of virulence genes, indicating a link between biofilm intensity and virulence gene expression.

Among the isolates, 5 XDR samples and 5 MDR samples exhibited strong biofilm formation, while 3 MDR samples showed medium biofilm formation and 2 MDR samples displayed weak biofilm formation. The ST associations of these isolates are detailed in Table 7.

## 4. Discussion

Bacteria deploy various mechanisms to enhance survival against destructive agents [35]. Among the most critical factors enabling *K. pneumoniae* to resist antibiotics, disinfectants, and antibacterial agents is the formation of biofilms [36]. This substance forms around the bacterial cell wall, providing a protective environment for the organism [37]. The antibiotic resistance pattern in *K. pneumoniae* isolates enables this bacterium to withstand treatment [38]. Our study revealed that cefepime had the highest prevalence of resistance, while amikacin had the lowest. These findings are consistent with those of other studies, such as Farhadi et al. (8% resistance) and Mokhtari et al. (14% resistance), which report low resistance to amikacin, making it a valuable option for treating *K. pneumoniae* infections in certain settings [39, 40]. On the other hand, our study identified a significantly higher resistance rate to imipenem (85.82%) compared to previous reports, such as Vaez, Ghalehnoo, and Yazdanpour (4%) [41]. In contrast, our study found a significantly higher resistance rate of 85.82%, which raises concerns about the widespread prevalence of carbapenem-resistant *K. pneumoniae* in hospital-acquired infections. Mokhtari et al. also found a high resistance rate to imipenem (98.3%) in their study, highlighting the increasing prevalence of carbapenem-resistant *K. pneumoniae*, a major concern in hospital-acquired infections [42]. The widespread resistance to carbapenems in *K. pneumoniae* is alarming, as these antibiotics are often the drugs of choice for severe infections caused by MDR and XDR strains [43].

In addition to imipenem, our study also examined other antibiotics from different classes, such as the third-generation cephalosporins and aminoglycosides. While resistance to cefepime was prevalent, resistance to amikacin remained low, as mentioned earlier, suggesting that aminoglycosides may still offer some efficacy against *K. pneumoniae* infections, especially those caused by strains with limited resistance profiles. However, the emergence of XDR *K. pneumoniae* strains, capable of resisting multiple classes of antibiotics, including beta-lactams, carbapenems, and aminoglycosides, makes treatment increasingly challenging [44]. The bacterium utilizes a variety of resistance mechanisms to counteract antibiotics, including the production of degrading enzymes, the activation of efflux pumps, the loss of porins to reduce drug uptake, and the modification of antibiotic targets [45]. Therefore, hospital-acquired infections caused by MDR *K. pneumoniae* isolates are associated with significant mortality and complications [37]. In our study, the prevalence of MDR isolates (53%) was lower compared to other reports, such as Malekjamshidi, which reported over 90%, and Kashefieh, with 85% MDR isolates [46, 47]. The prevalence of XDR isolates in our study (12%) was similar to that reported by Shadkam (12%) and Sharahi (10%) [25, 48]. However, Davoudabadi reported a higher prevalence of XDR isolates (35%) and also identified 7% of isolates as pan-drug resistance (PDR), indicating strains resistant to all tested antibiotics [49]. Variations in these resistance rates could be attributed to differences in antibiotic usage policies, infection control practices, geographical factors, study populations, and the source of the samples.

Biofilm is a significant factor enabling bacteria to resist a wide range of antibiotics, facilitating extensive and multidrug resistance, and enhancing survival. Our study found that out of 402 *K. pneumoniae* isolates from various clinical samples, 17 isolates (4.22%) had strong biofilms, 104 isolates (25.87%) had moderate biofilms, 180 isolates (44.77%) had weak biofilms, and 101 isolates (25.12%) did not form biofilms. There was a significant correlation between biofilm type and resistance, indicating that resistant and biofilm-producing bacteria are closely related [50, 51]. Shadkam et al. classified 25% as strong biofilm producers, 19% as moderate, 31% as weak, and 25% as nonbiofilm producers, with a higher strong biofilm rate than our study [25]. Karimi et al. reported that 20% of samples formed strong biofilms, higher than our study [52]. Haghighifar, Norouzi, and Dolatabadi found that out of 128 *K. pneumoniae* isolates, 86 (67.18%) had weak biofilms, 24 (18.75%) had moderate biofilms, and 18 (14.06%) had strong biofilms [53]. The significant correlation between biofilm formation and antibiotic resistance in *K. pneumoniae* highlights the necessity for targeted strategies to disrupt biofilm structures and combat resistant infections more effectively.

The prevalence of virulence genes among the 402 *K. pneumoniae* isolates in our study shows notable trends and comparisons to other studies. The high occurrence of *fimH1* (87.31%) and *mrkD* (98.50%) genes align with the findings from previous research, emphasizing their role in adherence and biofilm formation [54]. *FimH1* encodes for a Type 1 fimbrial adhesin, crucial for bacterial attachment to host tissues and biofilm formation [55]. *MrkD* encodes for a major pilin subunit of Type 3 fimbriae, also important in biofilm formation and host interaction. For instance, a study by Mohamed et al. reported similar high frequencies of these genes, indicating their ubiquitous presence in clinical isolates [56]. The *kpn* gene was found in 60.94% of our isolates, comparable to the 62.82% reported by Li et al. [57]. This consistency across different studies underlines the gene's significance in *K. pneumoniae* pathogenicity. The *kpn* gene is associated with the polysaccharide capsule of *K. pneumoniae*, a critical virulence factor that aids in evading the host immune response [58]. *YcfM*, present in 96.01% of our isolates, showed a higher prevalence than the 92.8% reported by Yousefi et al., suggesting a potential increase in the gene's distribution over time or variations in sample sources. *YcfM* is involved in lipopolysaccharide synthesis, contributing to the bacterium's outer membrane integrity and virulence [59]. The *entB* gene was present in all isolates, confirming its essential role in iron acquisition, which is critical for bacterial survival and virulence. *EntB* encodes for ENT synthase, a key enzyme in the biosynthesis of ENT, a high-affinity siderophore used by bacteria to scavenge iron from the host environment [60]. This finding is consistent with studies by Xiang et al., who also reported a 100% prevalence of *entB* in their isolates [61]. The *iutA* gene's presence in 45.27% of our isolates was higher compared to 21.4% reported by Sanikhani et al., indicating regional or environmental differences in the distribution of this virulence factor [62]. *IutA* encodes for an aerobactin receptor, another iron-chelating compound important for bacterial growth in iron-limited environments [63]. Genes associated with iron uptake, such as *irp-1* (37.56%), *irp-2* (33.83%), *ybtS* (63.18%), and *fyuA* (58.45%), exhibited variable frequencies. *Irp-1* and *irp-2* are components of the yersiniabactin biosynthetic cluster, *ybtS* is involved in the synthesis of yersiniabactin, and *fyuA* encodes a receptor for yersiniabactin [64]. Our findings for *ybtS* and *fyuA* are consistent with the 60% and 55% frequencies reported by Ballen et al., respectively, underscoring the critical role of these genes in iron acquisition and bacterial survival [65].

The discussion of our study highlights the prevalence of ST147 among the *K. pneumoniae* isolates, which is consistent with findings from a study conducted in Tehran by Shahkolahi et al. ST147 was associated with a higher presence of ESBL genes, indicating a potential threat to our region [66]. The second most prevalent ST in our study was ST11, which aligns with the results reported by Shahkolahi et al. Interestingly, we also identified ST15, with a frequency of 20%, which was not detected in the Tehran study, suggesting a greater genetic diversity among the isolates from Hamedan. A study by Davoudabadi et al. using MLST analysis found XDR-hvKP isolates belonging to ST15, ST377, ST442, and ST147, with ST147 being the only XDR type [49]. In our study, all five ST147 isolates were XDR, indicating a significant correlation between ST type and bacterial resistance, consistent with the findings of Shahkolahi et al., which reported the highest resistance genes in ST147. Furthermore, our study found that ST377 was MDR, while ST11 and ST15 were both MDR and XDR, suggesting that these STs can acquire resistance genes, leading to increased antibiotic resistance. Interestingly, all five ST147 isolates in our study formed strong biofilms and harbored a high number of virulence factor genes, while other STs did not exhibit strong biofilm formation. This observation suggests that ST147 resistance to a wide range of antibiotics and its ability to form strong biofilms might be attributed to the acquisition of numerous virulence factor genes. The high antibiotic resistance of ST147 may contribute to its dominance in antibiotic-treated patients, necessitating biofilm formation for survival.

## 5. Conclusion

Our study demonstrates the widespread prevalence of key virulence genes in *K. pneumoniae* isolates and their strong association with biofilm formation and antibiotic resistance. The genetic diversity observed through MLST underscores the complex epidemiology of *K. pneumoniae* infections. Continuous surveillance and molecular characterization are essential to understand the evolving virulence landscape and inform effective infection control strategies. The data also highlight the need for novel therapeutic approaches to combat the increasing challenge of MDR and biofilm-forming *K. pneumoniae*.

## Figures and Tables

**Table 1:** Primers used in the present study.

Target gene	Primers' sequences (5′ to 3′)	Product size (bp)	References
*fimH-1*	F-ATGAACGCCTGGTCCTTTGC	688	[17]
	R-GCTGAACGCCTATCCCCTGC		

*mrkD*	F-CCACCAACTATTCCCTCGAA	240	[30]
	R-ATGGAACCCACATCGACATT		

*kpn*	F-GTATGACTCGGGGAAGATTA	626	[17]
	R-CAGAAGCAGCCACCACACG		

*ycfM*	F-ATCAGCAGTCGGGTCAGC	160	[17]
	R-CTTCTCCAGCATTCAGCG		

*iroN*	F-AAGTCAAAGCAGGGGTTGCCCG	665	[31]
	R-GACGCCGACATTAAGACGCAG		

*entB*	F-ATTTCCTCAACTTCTGGGGC	371	[17]
	R-AGCATCGGTGGCGGTGGTCA		

*iutA*	F-GGCTGGACATCATGGGAACTGG	714	[17]
	R-CGTCGGGAACGGGTAGAATCG		

*fyuA*	F-GCGACGGGAAGCGATGATTTA	547	[17]
	R-TAAATGCCAGGTCAGGTCACT		

*irp-1*	F-TGAATCGCGGGTGTCTTATGC	238	[17]
	R-TCCCTCAATAAAGCCCACGCT		

*irp-2*	F-TCCCTCATAAAGCCCACGCT	287	[32]
	R-TCGTCGGGCAGCGTTTCTTCT		

*ybtS*	F-AGTGGTGCGTTCTGCGTC	477	[17]
	R-ATTTCTACATCTGGCGTTA		

*traT*	F-GGTGTGGTGCGATGAGCACAG	290	[17]
	R-CACGGTTCAGCCATCCCTGAG		

*rmpA*	F-ACTGGGCTACCTCTGCTTCA	535	[33]
	R-CTTGCATGAGCCATCTTTCA		

*magA*	F-GGTGCTCTTTACATCATTGC	1282	[34]
	R-GCAATGGCCATTTGCGTTAG		

*hlyA*	F-AACAAGGATAAGCACTGTTCTGGCT	1177	[17]
	R-ACCATATAAGCGGTCATTCCCGTCA		

*cnf-1*	F-AAGATGGAGTTTCCTATGCAGGAG	498	[17]
	R-CATTCAGAGTCCTGCCCTCATTATT		

**Table 2:** Antibiotic resistance, intermediate, and sensitivity profiles of *Klebsiella pneumoniae* isolates from women and men patients.

Antibiotic	Resistance status	Men (*n*, %)	Women (*n*, %)	Total (*n*, %)
Cefoxitin	Resistant	115 (61.83%)	127 (58.80%)	242 (60.19%)
	Intermediate	30 (16.13%)	33 (15.28%)	60 (14.93%)
	Sensitive	41 (22.04%)	56 (25.93%)	100 (24.88%)

Ciprofloxacin	Resistant	120 (64.52%)	148 (68.52%)	275 (68.40%)
	Intermediate	23 (12.37%)	17 (7.87%)	40 (9.95%)
	Sensitive	43 (23.11%)	51 (23.61%)	87 (21.64%)

Ceftazidime	Resistant	145 (77.96%)	160 (74.07%)	305 (75.87%)
	Intermediate	14 (7.53%)	11 (5.09%)	25 (6.22%)
	Sensitive	27 (14.52%)	43 (19.91%)	72 (17.91%)

Imipenem	Resistant	155 (83.33%)	190 (87.96%)	345 (85.82%)
	Intermediate	7 (3.76%)	8 (3.70%)	15 (3.73%)
	Sensitive	24 (12.90%)	18 (8.33%)	42 (10.45%)

Gentamicin	Resistant	132 (71.0%)	144 (66.67%)	276 (68.65%)
	Intermediate	21 (11.29%)	20 (9.26%)	41 (10.20%)
	Sensitive	33 (17.74%)	52 (24.07%)	85 (21.14%)

Amikacin	Resistant	80 (43.01%)	85 (39.35%)	165 (41.04%)
	Intermediate	21 (11.29%)	16 (7.41%)	37 (9.20%)
	Sensitive	85 (45.70%)	115 (53.24%)	194 (48.26%)

Piperacillin–tazobactam	Resistant	38 (20.43%)	37 (17.13%)	75 (18.65%)
	Intermediate	12 (6.45%)	17 (7.87%)	29 (7.21%)
	Sensitive	136 (73.12%)	162 (75.00%)	298 (74.13%)

Ampicillin–sulbactam	Resistant	62 (33.33%)	60 (27.78%)	122 (30.34%)
	Intermediate	23 (12.37%)	25 (11.57%)	48 (11.94%)
	Sensitive	101 (54.30%)	131 (60.65%)	232 (57.71%)

Trimethoprim–sulfamethoxazole	Resistant	124 (66.67%)	128 (59.26%)	252 (62.68%)
	Intermediate	16 (8.60%)	14 (6.48%)	30 (7.46%)
	Sensitive	46 (24.73%)	74 (34.26%)	120 (29.85%)

Cefepime	Resistant	167 (89.78%)	189 (87.50%)	356 (88.55%)
	Intermediate	—	—	—
	Sensitive	19 (10.21%)	27 (12.5%)	46 (11.44%)

**Table 3:** Prevalence of MDR and XDR *Klebsiella pneumoniae* according to age group.

Age group	Number of MDR isolates (%)	*p* value	Number of XDR isolates (%)	*p* value	Total (%)
0–5	22 (62.85)	0.045	7 (20.00)	0.081	35 (87.06)
6–15	18 (40.0)	0.135	3 (6.66)	0.131	45 (11.19)
16–30	29 (53.70)	0.146	6 (11.11)	0.468	54 (13.43)
31–45	24 (48.00)	0.46	4 (8.00)	0.202	50 (12.43)
46–60	68 (50.00)	0.002	19 (13.97)	0.035	136 (33.83)
> 60	53 (64.63)	< 0.001	14 (17.07)	0.032	82 (20.39)

**Table 4:** Prevalence of virulence genes in *Klebsiella pneumoniae* isolates from women and men patients.

Virulence gene	Number of positive isolates			Percentage of positive isolates		
	Women	Men	Total	Women (%)	Men (%)	Total (%)
*fimH1*	189	162	351	86.11	87.09	87.31
*mrkD*	216	180	396	100.00	96.77	98.50
*kpn*	136	109	245	62.96	58.60	60.94
*ycfM*	211	175	386	97.68	94.08	96.01
*entB*	216	186	402	100.00	100.00	100.00
*iutA*	101	81	182	46.75	43.54	45.27
*irp-1*	79	72	151	36.57	38.70	37.56
*irp-2*	75	61	136	34.72	32.79	33.83
*ybtS*	151	103	254	69.90	55.37	63.18
*fyuA*	141	94	235	65.27	50.53	58.45
*traT*	78	73	151	36.11	39.24	37.56
*rmpA*	24	21	45	11.11	11.29	11.19
*magA*	8	3	11	3.70	1.61	2.73
*iroN*	2	0	2	0.92	0.00	0.49
*hylA*	0	0	0	0.00	0.00	0.00
*cnf-1*	0	0	0	0.00	0.00	0.00

**Table 5:** Comparison of biofilm phenotypic patterns in clinical isolates of MDR and XDR.

	Number	Strong biofilm	Moderate biofilm	Weak biofilm	Biofilm formation	No biofilm	*p* value
MDR	214	1	58	97	156	58	0.341
XDR	53	16	35	2	53	0	< 0.001
Non-MDR/non-XDR	135	0	11	81	92	43	—

**Table 6:** Comparison of virulence genes in biofilm-forming clinical isolates of MDR and XDR.

Gene	Strong biofilm	Moderate biofilm	Weak biofilm	Biofilm formation	Nonbiofilm	*p* value	MDR	Non-MDR/non-XDR	*p* value	XDR	*p* value
*fimH1*	17	91	152	260	91	0.772	214	84	< 0.001	53	< 0.001
*mrkD*	17	104	180	301	95	< 0.001	214	129	< 0.01	53	< 0.001
*kpn*	17	92	98	207	38	< 0.001	154	46	< 0.001	45	< 0.001
*ycfM*	17	104	175	296	90	< 0.001	189	135	< 0.001	52	< 0.001
*entB*	17	104	180	301	101	< 0.01	214	135	< 0.001	53	< 0.001
*iutA*	17	86	61	184	18	< 0.001	107	25	< 0.001	50	< 0.001
*irp-1*	15	67	57	139	12	< 0.001	56	48	< 0.05	47	< 0.001
*irp-2*	12	56	55	123	40	0.932	54	37	> 0.05	45	< 0.001
*ybtS*	11	79	97	187	67	0.676	195	33	< 0.001	53	< 0.001
*fyuA*	15	63	69	147	88	< 0.001	147	38	< 0.001	50	< 0.001
*iroN*	1	1	0	2	0	0.405	0	0	—	2	< 0.05
*traT*	16	49	57	122	29	0.021	97	24	< 0.001	30	< 0.001
*rmpA*	15	11	15	41	3	< 0.01	25	6	< 0.05	14	< 0.001
*hylA*	0	0	0	0	0	—	0	0	—	0	—
*magA*	8	3	0	11	0	0.048	5	4	> 0.05	2	> 0.05
*cnf-1*	0	0	0	0	0	—	0	0	—	0	—

**Table 7:** Relationship between biofilm formation, antibiotic resistance, and sequence type.

Sequence type (Pasteur scheme)	Number	Allelic profile (*gapA*, *infB*, *mdh*, *pgi*, *phoE*, *rpoB*, *tonB*)	XDR	MDR	Strong biofilm	Moderate biofilm
ST147	5	3 + 4 + 6 + 1 + 7 + 4 + 38	5	0	5	0
ST11	3	3 + 3 + 1 + 1 + 1 + 1 + 4	0	3	0	3
ST15	2	1 + 1 + 1 + 1 + 1 + 1 + 1	0	2	0	2

Abbreviation: ST = sequence type.

## Data Availability

All data generated or analyzed during this study are included in this article. The raw data are available from the corresponding author upon reasonable request.

## Supporting Information

Supporting File 1: Results of gel electrophoresis for PCR analysis, showing the amplification of *iutA* and *mrkD* genes.
