# Supplementary figures and images for: Comprehensive Analysis of Virulence Genes, Antibiotic Resistance, Biofilm Formation, and Sequence Types in Clinical Isolates of Klebsiella pneumoniae
Source: Can J Infect Dis Med Microbiol. 2024 Dec 19;2024:1403019. doi: 10.1155/cjid/1403019 (PMC11671628; doi:10.1155/cjid/1403019)

PC NC 1 2 3 4 5 6 7 8 9

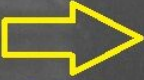  
*mrkD* 240 bp

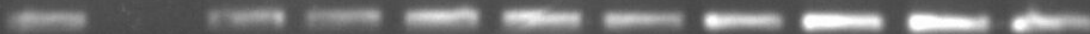

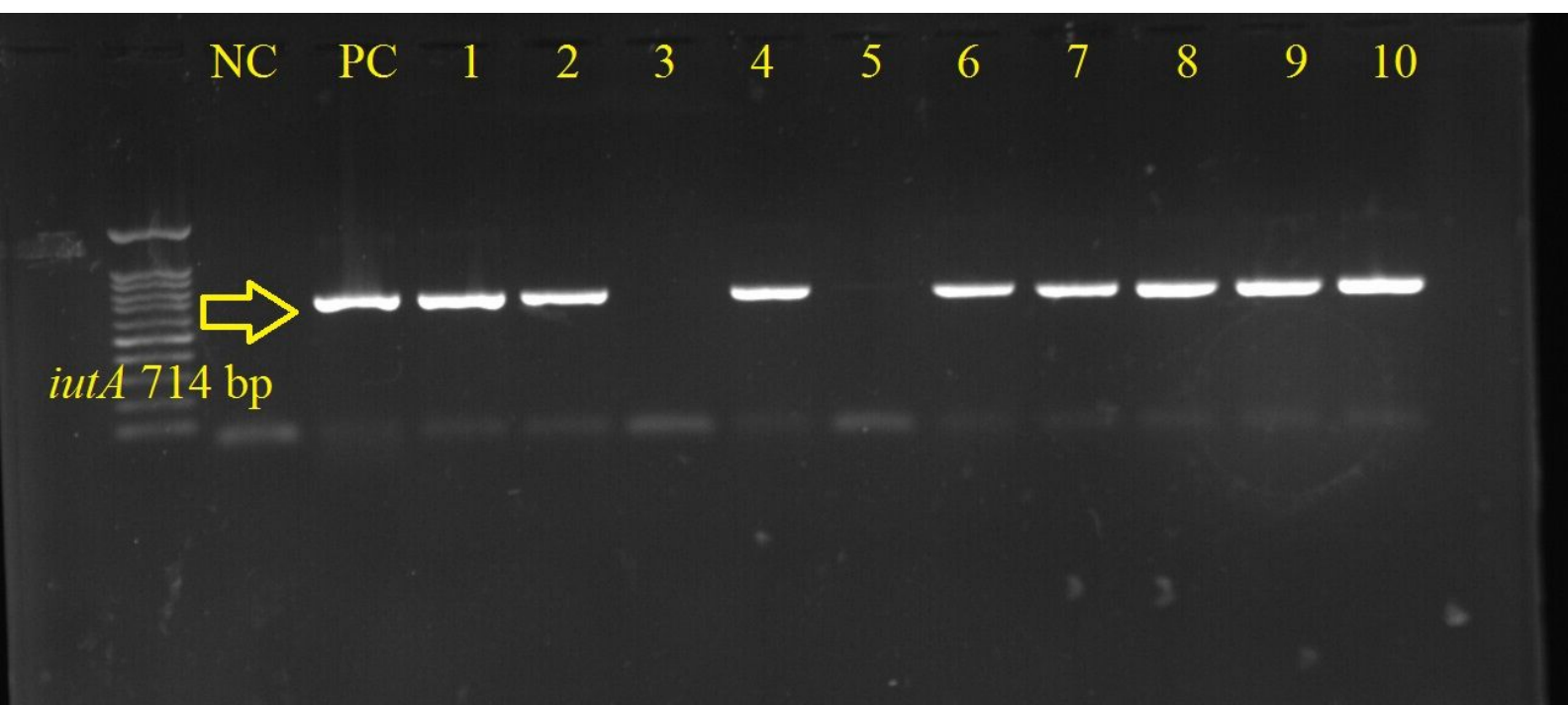

Supplement: Supporting Information — Additional supporting information can be found online in the Supporting Information section. [file 1403019.f1.pdf]
